# Diagnosis and treatment of congenital abdominal aortic aneurysm: a systematic review of reported cases

**DOI:** 10.1186/s13023-015-0225-x

**Published:** 2015-01-21

**Authors:** Yamei Wang, Yuhong Tao

**Affiliations:** Department of Pediatrics, West China Second University Hospital, Sichuan University, No.20, Section 3, Renmin Nan Lu, Chengdu, Sichuan Province 610041 China; Key Laboratory of Obstetric & Gynecologic and Pediatric Diseases and Birth Defects of Ministry Education, West China Second University Hospital, Sichuan University, Sichuan, China

**Keywords:** Aortic aneurysms, Abdominal, Congenital, Ultrasonography, Surgical repair, Children

## Abstract

**Background:**

Congenital abdominal aortic aneurysm (AAA) is distinctly rare in infants and children and carries a high mortality rate. Our objective was to summarize the experience of the diagnosis and treatment in patients with congenital AAA.

**Methods:**

Reported cases of congenital AAA published prior to November 8, 2014, were identified through PubMed, EMBASE, Web of Science, and reference lists. All selected cases were evaluated for main clinical characteristics.

**Results:**

Twenty-six cases of congenital AAA were identified in the English language literature. Congenital AAA occurred primarily in children under three years old, but it was also found in young adults and fetuses. With regards to the localization, the great majority of congenital AAA was infrarenal AAA. The majority of the AAA patients lacked specific symptoms, and a painless pulsatile abdominal mass was the most common clinical presentation. The diagnosis of AAA was based on ultrasound scanning in twenty-five cases, multi-slice spiral computed tomography angiography (MSCTA) in sixteen cases, and magnetic resonance angiography (MRA) in nine cases. Histopathological analyses were available in seven cases. Seven patients received conservative management. Surgical treatment was performed in seventeen cases, and open repair with an artificial graft was the main surgical intervention. The mortality associated with congenital AAA was high (30.76%). Ruptured aneurysm and renal failure were the main causes of death.

**Conclusions:**

Good outcomes can be achieved in children with early identification of congenital AAA and individualized surgical repair with grafts.

## Background

Abdominal aortic aneurysm (AAA) is the pathologic local dilation of the abdominal aorta and is defined as an aorta size of more than 30 mm or a local dilation of the abdominal aorta greater than 50% compared with another site along the aorta. Despite the evolution of the understanding and treatment of AAA over the past few decades, it continues to be a major threat to health due to the 80% overall mortality in the event of rupture.

Acquired AAA is associated with various definitive predisposing factors, including infection (e.g., bacterial, tuberculosis and fungal infection) [1], congenital connective tissue disease (e.g., Marfan’s syndrome, Ehlers-Danlos syndrome, and tuberous sclerosis) [2,3], trauma (e.g., umbilical artery catheterization) [4], and vasculitis (e.g., Takayasu arteritis, polyarteritis nodosa, giant cell arteritis, and Kawasaki’s syndrome) [5]. These etiologies may cause the denaturation of the vessel wall, extensive infiltration of monocytes, lymphoid cells and macrophages, increased vessel wall stress, blood flow disorders and a decrease in the nourishment of blood vessels, which leads to the expansion of the abdominal aortic wall and ultimately results in AAA.

In comparison with acquired AAA, congenital AAA has an unknown etiology. Congenital AAA in neonates, infants and children is extremely rare and carries a high mortality rate. To date, fewer than thirty pediatric cases have been reported. Awareness of the diagnosis and advances in diagnostic techniques may allow the diagnosis to be made earlier in the course of disease. Improved operative skills and conservative treatment may significantly decrease the mortality rate. To summarize the experience of the diagnosis and treatment in patients with congenital AAA, a systematic review of cases published in the literature was conducted.

## Methods

### Sources

All English publications that reported cases of congenital AAA in any population were assessed. A comprehensive search of PubMed, EMBASE, and Web of Science was performed for all relevant papers published prior to November 8, 2014. The search keywords included (abdominal aortic aneurysm and congenital) OR (aneurysms, abdominal aortic and congenital). Language limits for all searches were set to English. A manual search was performed by checking the reference lists of the original reports and review articles that were retrieved through the electronic searches to identify studies not yet included in the computerized databases.

### Study selection

All potentially relevant papers were reviewed independently by the authors. We assessed the eligibility of each article and abstracted data with standardized data abstraction forms. Disagreements were resolved through discussion.

**Figure 1:**
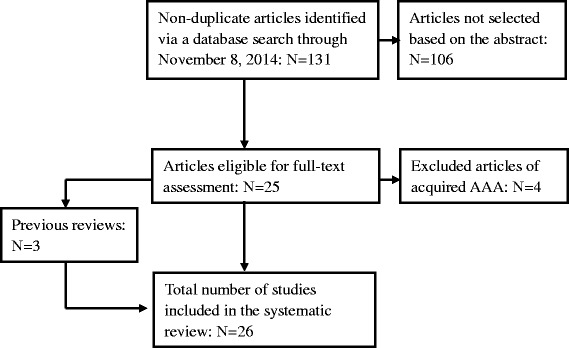
**Flow chart of the systematic review.**

First, all non-duplicate publications reporting cases of congenital AAA were selected for review. Altogether, 131 articles were identified from 1967 to November 8, 2014. Case reports and reviews relative to congenital AAA were screened to select eligible articles. Next, all four articles regarding acquired AAA were excluded. Finally, the reference lists of all three previous reviews concerning congenital AAA were reviewed to search for additional eligible articles. Twenty-six articles were considered eligible after this process. The results of the review process are outlined in Figure 1.

All selected cases were evaluated for the main clinical characteristics. The following data were collected from all eligible articles: authors, age at discovery, gender, clinical presentation, diagnostic methods, treatment and outcome.

## Results

Twenty-six cases of congenital AAA were identified in the English language literature [6-31]. The main clinical characteristics of these cases are summarized in Table 1.

**Table 1:** **Main clinical characteristics of twenty-six previously reported cases of congenital AAA**

**Author**	**Age at discovery**	**Gender**	**Location**	**Surgical treatment**	**Outcome**
**Howorth** **[** 6 **]**	1 day	Female	Infrarenal	Exploratory laparotomy	Rupture and death during operation
**Darden WA** **[** 7 **]**	2.5 years	Male	Infrarenal	Dacron aortic graft	None reported
**Sterpetti AV** **[** 8 **]**	19 years	Male	Infrarenal	Dacron aortic graft	None reported
**Odagiri S** **[** 9 **]**	1 year	Male	Infrarenal	Dacron aortic graft	Healthy at 10 months after surgery
**Latter D** **[** 10 **]**	1 month	Male	Infrarenal	8 mm polytetrafluoroethylene tube graft	Healthy
**Saad SA** **[** 11 **]**	6 weeks	Male	Infrarenal	Aneurysmorrhaphy	Healthy at 3 months after surgery
**Myrmel T** **[** 12 **]**	30 years	Male	Infrarenal	None reported	None reported
**Sarkar R** **[** 13 **]**	4 years	Female	Infrarenal	Aneurysmorrhaphy	None reported
**Malee MP** **[** 14 **]**	32 weeks’ gestation	Female	Juxtarenal	None	Died of acute pulmonary hypertension and cardiac dysfunction at age 9 days
**Kim ES** **[** 15 **]**	7 days	Female	Juxtarenal	None	Died of cardiac and renal failure at age19 days
**Mehall JR** **[** 16 **]**	6 weeks	Male	Juxtarenal	Bifurcated 7.4 mm Gore-Tex graft	Healthy at 1 month after surgery
**Laing AJ** **[** 17 **]**	1 year	Male	Infrarenal	Exploratory laparotomy	Rupture and death during operation
**Dittrick K** **[** 18 **]**	12 years	Male	Infrarenal	Dacron tube graft	Healthy at 2 years after surgery
**Bell P** **[** 19 **]**	1 day	Female	Infrarenal	Cryopreserved allograft (CryoLife)	Healthy at 14 months after surgery
**Cheung SC** **[** 20 **]**	6 months	Male	Juxtarenal	None	Thrombosis of the aneurysm and renal dysfunction at 3 years
**Buddingh KT** **[** 21 **]**	1 day	Male	Infrarenal	None	Healthy at 7 months, aneurysm grown to 9.3 mm max diameter
**Kim JI** **[** 22 **]**	21 weeks’ gestation	None reported	Infrarenal	Dacron aortic graft	Uneventful post-operation recovery
**Malikov S** **[** 23 **]**	28 weeks’ gestation	Male	Juxtarenal	Repair with native iliac vessels	Healthy at 39 months after surgery
**Cantinotti M** **[** 24 **]**	22 weeks’ gestation	None reported	Infrarenal	None reported	None reported
**Tsunematsu R** **[** 25 **]**	25 weeks’ gestation	Male	Unspecified	None	Stable after 6 months
**McAteer J** **[** 26 **]**	34 weeks’ gestation	Male	Thoracoabdominal	None	Died of rupture at age 28 days
**Cho YP** **[** 27 **]**	23 months	Male	Infrarenal	Cryopreserved cadaveric artery	Healthy at 10 months after surgery
**Meyers RL** **[** 28 **]**	Neonate	None reported	Infrarenal	Decellularized, antigen-reduced cryopreserved allograft	Healthy at 29 months after surgery
**Ko Y** **[** 29 **]**	2 months	Male	Thoracoabdominal	Dacron aortic graft	Healthy
**Fettah ND** **[** 30 **]**	1 day	Female	Infrarenal	Repair with polytetrafluorethylene	Died of sepsis and cardiopulmonary insufficiency at age 5 weeks
**Bivins HA** **[** 31 **]**	19 weeks’ gestation	Male	Infrarenal	None	Died of renal failure at age 12 days

Congenital AAA mainly occurred in neonates and infants (57.69%), but it was also found in young adults and fetuses (42.31%). Of these twenty-six cases, seven were diagnosed prenatally, six were diagnosed as neonates, nine were diagnosed between one month and three years of age, two were diagnosed between three and twelve years of age, one was diagnosed at age nineteen, and one was diagnosed at age thirty. As shown in Table 1, seventeen of the patients were male, six were female, and the gender of the remaining three patients was not reported.

With regards to localization, the great majority of the congenital AAA cases were infrarenal AAA (69.23%). Of the twenty-six cases, eighteen occurred infrarenally, five occurred juxtarenally, two occurred thoracoabdominally, and the remaining case was unspecified. These aneurysms ranged from 1.6–11.0 cm in maximum diameter and extended to one or both of the iliac arteries in the pelvis. In four cases, additional aneurysms were identified in the iliac artery, renal artery, or superior mesenteric artery [11,15,16,20].

Clinical presentation varied from a complete lack of symptoms to ruptures. A pulsatile abdominal mass was observed on examination or unexpectedly. Of the twenty-six cases, six were asymptomatic, and three were discovered during surgical procedures to address other diseases. Other presentations included painless pulsatile abdominal mass in seven cases [6,10,11,15,16,19,26], emesis in two cases [16,19], respiratory distress in two cases [9,15], failure to pass meconium in one case [6], paleness and shock in one case [17], and vomiting and irritability in one case [27]. Five patients showed concomitant disorders, including Wilms’ tumor [6], nesidioblastosis [11], infantile hypertrophic pyloric stenosis [16], renal dysplasia [20], and porencephaly [25].

Imaging examination was the main method for the diagnosis of AAA. The diagnosis of AAA was made by ultrasound scanning in twenty-five cases, MSCTA in sixteen cases, and MRA in nine cases. In one case, an intra-abdominal mass was found in an abdominal X-ray plain film, but AAA was only confirmed during the operation. Histopathological analyses were available in seven cases [6,9,14-17,19]. The most striking histopathological changes of congenital AAA were observed in the intima. These changes included calcifications, thrombosis, and ulcerations and ruptures of the layers. The aneurysm walls differed in the distribution of elastin, the presence of fibrosis, and the thickness of the vessel layers.

Seven patients received conservative management because of extensive aneurysms, small vessel size, and hemodynamic instability. Seventeen patients underwent surgical treatment (65.38%). The timing of these repairs ranged from the neonatal period to twelve years of age. Aneurysmorrhaphy was used in two cases, and good results were noted three months after the operation in one case. Aortobiliac reconstruction using native vessels was performed in one case. In nine cases, the aneurysms were successfully reconstructed with an artificial graft. In three cases, they were reconstructed with an allograft. The maximum time of follow-up in these cases was fourteen months. It was still impossible to assess the value of this surgical repair because there was a limited amount of information regarding the patency of the graft, necessary reoperation, or long-term survival of the patients.

The mortality of congenital AAA was high (30.76%). Ruptured aneurysm and renal failure were the main causes of death. Offering timely elective repair remains the most reliable strategy for preventing death. Of the seven patients who underwent conservative management, one patient died of acute pulmonary hypertension and cardiac dysfunction, one died of cardiac and renal failure, one died of rupture, one died of thrombosis of the aneurysm and renal dysfunction at three years, one died of renal failure, and the remaining two patients were stable after six to seven months of follow-up. Of the seventeen surgical patients, eleven underwent successful repair and had uneventful recoveries, two newborns did not survive operative intervention, three had unknown outcomes, and one died of sepsis and cardiopulmonary insufficiency.

## Discussion

### Etiology

According to the Hamburg Classification of Congenital Vascular Malformations [32], congenital AAA is defined as a localized truncular form of arterial defect. This arterial malformation is the result of a developmental arrest occurring along the arterial system in the later stage of embryogenesis [33]. Currently, little is known about the molecular and genetic origins of congenital AAA due to the rarity of this disease.

The etiology of congenital AAA may be associated with a molecular genetic defect. The transforming growth factor-β (TGF-β) signaling pathway controls cellular proliferation, growth and differentiation and regulates several functions of the connective tissue. Disruption of genes coding for components of the TGF-β signaling pathway or its interactors, such as fibrillin-1, have recently been described in patients with aortic aneurysm. Marfan’s syndrome and Loeys-Dietz syndrome represent rare congenital connective tissue disorders that have similar patterns of cardiovascular defects (e.g., thoracic aortic aneurysm); these syndromes are caused by mutations in the genes encoding for TGF-β2 or TGF-β receptor (TGFBR) I or II [34-36]. These molecular and genetic processes are of great significance for investigating the etiology of congenital AAA. Unfortunately, these genetic defects were not described in previous patients with congenital AAA. Therefore, the relationship between these genetic defects and congenital AAA remains unclear. Further study will be needed to demonstrate their true relationship.

### Signs and symptoms

There is no specificity in the clinical manifestation of congenital AAA. The clinical presentation of congenital AAA varies considerably according to the vessel affected. In the present review, many cases presented with a painless pulsatile abdominal mass, which drew much attention. As an AAA expands, it may become painful. Renovascular hypertension and renal failure should be highly concerning. These presentations were found to be the result of renal aneurysms, renal artery thrombosis or renal dysplasia.

### Diagnosis and differential diagnosis

Congenital AAA can usually be diagnosed with specific imaging modalities. The primary cause of misdiagnosis is doctors not considering the possibility of an AAA. Once a clinical presentation such as abdominal pulsatile mass, abdominal vascular murmur, or tremors is detected, AAA should be considered. Relevant imaging such as ultrasonography, MSCTA and MRA should be performed to identify AAA. Ultrasonography can be used to screen for aneurysms and to determine their size. MSCTA scanning has approximately 100% sensitivity for aneurysms and is useful for preoperative planning because it can detail the anatomy and the possibility of endovascular repair. In the case of suspected rupture, it can also reliably detect retroperitoneal fluid. The present review also indicates that additional aneurysms and concomitant diseases are common. These conditions are occasionally critical for determining the optimal time for surgical repair. Thus, comprehensive evaluation with multimodality imaging is recommended for investigating patients with congenital AAA.

Of course, it is very important to note that various cases of acquired AAA are far more frequent than cases of congenital AAA. The secondary causes, including congenital connective tissue disorder (e.g., Marfan’s syndrome and Ehlers-Danlos syndrome), must be ruled out for a definitive diagnosis of congenital AAA. A recent study also suggested that some patients diagnosed with congenital AAA might be suffering from variants of Ehlers-Danlos syndrome, with the most likely variant being type IV [37]. This condition is due to abnormal type III procollagen present in the wall of arteries. Patients with Ehlers-Danlos syndrome (type IV) do not have excessive skin laxity, and joint hypermobility is minimal, which means that the clinical diagnosis can be easily missed. Thus, skin biopsy and collagen III analysis are necessary to confirm the diagnosis.

### Management and Treatment

Current therapeutic decisions are largely extrapolated from the adult literature, and there is no universal approach to the management of congenital AAA. The treatment options include conservative management, surveillance leading to eventual repair and surgical repair.

### Conservative management

The conservative management of AAA has, to a large degree, been ignored until recently. Conservative management aims to prevent AAA progression and rupture, reduce the long-term requirement for surgery and limit cardiovascular events, and provide an important supplement to surgical treatment. Conservative management is indicated for patients for whom surgical repair carries a high risk of mortality and is unlikely to improve life expectancy [38]. In the present review, five patients with renal involvement received only conservative management due to the presence of concomitant renal anomalies.

The conservative measures include ultrasound follow-up and internal medical therapy such as statins, beta-receptor blockers, matrix metalloproteinase inhibition, angiotensin converting enzyme inhibitors (ACEI), anti-platelet drugs, and non-steroidal anti-inflammatory drugs (NSAIDs). Hypertension must be controlled because it may exacerbate the stress on the aortic wall, and antihypertensive therapy can decrease this stress [39]. This stress can be relieved with drugs such as ACEI. In the present review, calcium channel blockers were used in two cases of congenital AAA. However, the efficacy of such drugs in pediatric AAA management has not been investigated [38]. The natural history of AAA is frequently characterized by the development of a mural thrombus [40]. These thrombi contain large numbers of inflammatory cells, particularly neutrophils, and high concentrations of pro-inflammatory cytokines and proteolytic enzymes. The growth of a mural thrombus is also a better predictor of rupture than arterial diameter in cases of AAA [41]. There has been one case report of parietal thrombus in congenital AAA [30]. In this condition, AAA should be closely monitored via multimodal imaging until surgical repair is performed [42]. In a rat model of AAA, platelet inhibition has been found to limit AAA development [43]. However, the efficacy of anti-platelet medication in limiting congenital AAA progression has not been demonstrated [38].

### Surgery

There are currently two modes of repair available for an AAA: open repair and endovascular aneurysm repair (EVAR). However, treatment of congenital AAA in a neonate, infant, or young child requires additional considerations. Although experience with EVAR in adults has been increasing rapidly [44], there are no reported cases of the use of these devices in children with congenital AAA. Thus, EVAR is not feasible in infants or children. Although no definite size criteria for the surgical repair and no standard operative approach have been established, open repair with a graft remains the main surgical intervention.

Aneurysmorrhaphy increases the risk of recurrence because it maintains the dysplastic aortic segment. In the present review, aneurysmorrhaphy was only used in two cases. It was reported that an artificial graft was an appropriate vascular substitute in children and did not impact their growth and development or their quality of life [45,46]. However, the use of an artificial graft, particularly in newborns, raises the potential risk of mismatching between the fixed graft diameter and the growing native vessel diameter. Graft replacement is required as the children grow. The patency of a synthetic vascular graft with a diameter of less than 6 mm is poor. It may be more appropriate to delay surgery for smaller aneurysms to produce better results and prevent the need for follow-up surgery [47]. Despite this issue, the use of an artificial graft was the most commonly used treatment in the present review.

The use of a standard cryopreserved allograft is not recommended because they induce an immunologic response that has been linked to a high incidence of fibrosis, calcification, and degradation. Recently, a new generation of decellularized cryopreserved allograft with reduced immunogenicity has been introduced for the treatment of congenital heart disorders [48]. These grafts appear to promote long-term patency and to be well adapted to growth in children. One report of decellularized branched pulmonary allograft for aortoiliac reconstruction yielded good results [28]. However, it is still impossible to assess the value of this surgical repair for cases of congenital AAA because there is limited information regarding the patency of the graft, necessary reoperation, and long-term survival of the patients.

In the present review, reconstruction of AAA was achieved using native vessels without exogenous material in a 10-day-old boy [23]. This method was feasible, allowed natural vessel growth, and avoided graft-related complications.

### Unresolved questions

The cause of congenital AAA remains unclear, and little is known about the molecular pathways leading to AAA formation. In the future, the genetics of AAA, the mode of inheritance, the candidate gene approach and linkage analysis may illuminate the genetic background of congenital AAA.

## Conclusions

Good outcomes can be achieved in children with early identification of congenital AAA and individualized surgical repair with grafts. Once suggestive clinical presentation such as pulsatile abdominal mass are observed, multimodality imaging such as ultrasonography and MSCTA should immediately be performed to confirm the diagnosis of AAA. When AAA is encountered, an exhaustive search for the underlying factors should be conducted to exclude acquired AAA. Management strategies should be highly individualized, and early individualized surgical repair with grafts is advocated for the treatment of congenital AAA.

## Abbreviations

AAA: Abdominal aortic aneurysm
MSCTA: Multi-slice spiral computed tomography angiography
MRA: Magnetic resonance angiography
TGF-β: Transforming growth factor-β
ACEI: Angiotensin converting enzyme inhibitors
NSAIDs: Non-steroidal anti-inflammatory drugs
EVAR: Endovascular aneurysm repair
